# In Vitro Responses of Plant Growth Factors on Growth, Yield, Phenolics Content and Antioxidant Activities of *Clinacanthus nutans* (Sabah Snake Grass)

**DOI:** 10.3390/plants9081030

**Published:** 2020-08-14

**Authors:** Zainol Haida, Jaafar Juju Nakasha, Mansor Hakiman

**Affiliations:** Department of Crop Science, Faculty of Agriculture, Universiti Putra Malaysia, UPM, Serdang 43400, Selangor, Malaysia; haidacrzainol@gmail.com (Z.H.); jujunakasha@upm.edu.my (J.J.N.)

**Keywords:** *Clinacanthus nutans*, cytokinin, auxin, basal medium strength, sucrose concentration, antioxidant

## Abstract

*Clinacanthus nutans*, commonly known as Sabah snake grass, is one of the more important medicinal plants in Malaysia’s herbal industry. *C. nutans* has gained the attention of medical practitioners due to its wide range of bioactive compounds responsible for various biological activities, such as anti-cancer, anti-venom and anti-viral activities. Due to its high pharmacological properties, the species has been overexploited to meet the demands of the pharmaceutical industry. The present study was conducted to establish a suitable in vitro culture procedure for the mass propagation of *C. nutans.* Murashige and Skoog (MS) basal medium, supplemented with different types of cytokinins, auxins, basal medium strength and sucrose concentrations, were tested. Based on the results, a full-strength MS basal medium supplemented with 12 µM 6-benzylaminopurine (BAP) and 30 g/L sucrose was recorded as the best outcome for all the parameters measured including the regeneration percentage, number of shoots, length of shoots, number of leaves and fresh weight of leaves. In the analysis of the phenolics content and antioxidant activities, tissue-cultured leaf extracts assayed at 100 °C exhibited the highest phenolic content and antioxidant activities. The propagation of *C. nutans* via a plant tissue culture technique was recorded to be able to produce high phenolic contents as well as exhibit high antioxidant activities.

## 1. Introduction

Medicinal plants, also referred to as herbal plants, have been used for centuries as a source for alternative medicines in the prevention and treatments of various diseases. Globally, plant-based products have been well-accepted due to scientific evidence that proved their low to no side effects and low price compared to modern medicine [1]. Presently, approximately 70–80% of the world’s population depend on plant-based products as their primary healthcare needs [2]. In Malaysia, the herbal industry has become one of the most important sectors with RM 80 million (USD 18 million) production values in 2018. With an ever increasing demand, the value has been projected to increase manifolds [3].

*Clinacanthus nutans*, commonly known as Sabah snake grass, is a medicinal plant species belonging to the family Acanthaceae and is among the more important medicinal plants in Malaysia. It is an annual shrub which grows up to three meters tall with lanceolate-ovate shaped leaves [4]. The species is being widely used in Southeast Asian countries like Malaysia, Thailand and Indonesia as a remedy for the treatment of herpes simplex virus, varicella-zoster virus, fever, diabetes and as anti-venom for snake and scorpion bites [5,6]. Dried leaves of *C. nutans* are also known to treat hepatitis infection, fever, eczema, shingles and inflammation [6,7,8,9]. The medicinal properties of *C. nutans* leaf extracts have been recognized for many generations and have been commonly used in traditional medicines. According to the Ministry of Agriculture and Agro-based Industry Malaysia, *C. nutans* has been selected under the National Key Economic Area (NKEA) research grant schemes alongside 17 other herbal plants. They were selected based on their potentials for commercialization [10]. Meanwhile, in Thailand, the National Drug Committee recognized *C. nutans* as one of Thailand’s National List of Essential Medicines in 2011 [11].

Presently, the extraction of bioactive compounds or plant secondary metabolites from plant-based sources has gained attention in pharmaceutical industries. Advances in plant biochemistry and herbal medicines have identified several bioactive compounds in *C. nutans* leaf extracts, namely stigmasterol, kaempferol, quercetin, gallic acid, apigenin and vitexin. Additionally, several unique bioactive compounds have also been identified such as clinacoside (A, B, C), cycloclinacoside (A1, A2), clinamide (A, B, C, D, E), 2-cis-entadamide A and entadamide (A, C) [8,12,13].

Generally, natural products are the most preferred source of antioxidants in disease prevention than synthetic antioxidants due to them having few side effects. The demand for the raw material of *C. nutans* has led to the over-harvesting of the wild species which has significantly reduced their availability in nature [4]. Hence, a biotechnological approach in the propagation of the plant through an in vitro technique is perceived as an alternative method for the large-scale production of the raw materials in a shorter time. Plant growth regulators, the basal medium strength and sucrose concentration largely influence the success of an in vitro technique for plant propagation. Through this technique, the phenolic contents and antioxidant activities are also known to be affected. The present study was undertaken to investigate the effects of plant growth regulators, basal medium strength and sucrose concentrations of in vitro culture of *C. nutans* with the aim of optimizing the basal medium for the mass propagation of plants. The study also was conducted to analyze the phenolic contents and antioxidant activities between the leaves of tissue-cultured and conventionally propagated *C. nutans* as affected by different aqueous extraction temperatures.

## 2. Results

### 2.1. In Vitro Propagation of Clinacanthus nutans via Single Node Culture

#### 2.1.1. Shoot Induction of *C. nutans* as Affected by Cytokinins

In plant tissue culture techniques, plant growth regulators such as cytokinins and auxins are vital elements directly affecting plant growth and the development of the plantlets. Cytokinin is commonly used in shoot induction and the multiplication stages of explants. In the present study, the shoot induction of nodal segments was conducted by supplementing Murashige and Skoog (MS) [14] basal medium with various concentrations of 6-benzylaminopurine (BAP), a synthetic cytokinin, and N6-furfuryladenine (kinetin) (Table 1). The results indicated that the MS basal medium without cytokinin (MS0), 4 µM BAP and 4 µM kinetin exhibited 88.89% shoot regeneration. Other treatments were successful in exhibiting 100% shoot regeneration. Shoot induction was observed from day four after explant inoculation. During shoot regeneration, the enlargement of the dormant axillary buds of nodal segments was observed, followed by bud breaks. Subsequently, shoot formation and proliferation occurred.

**Figure 1:**
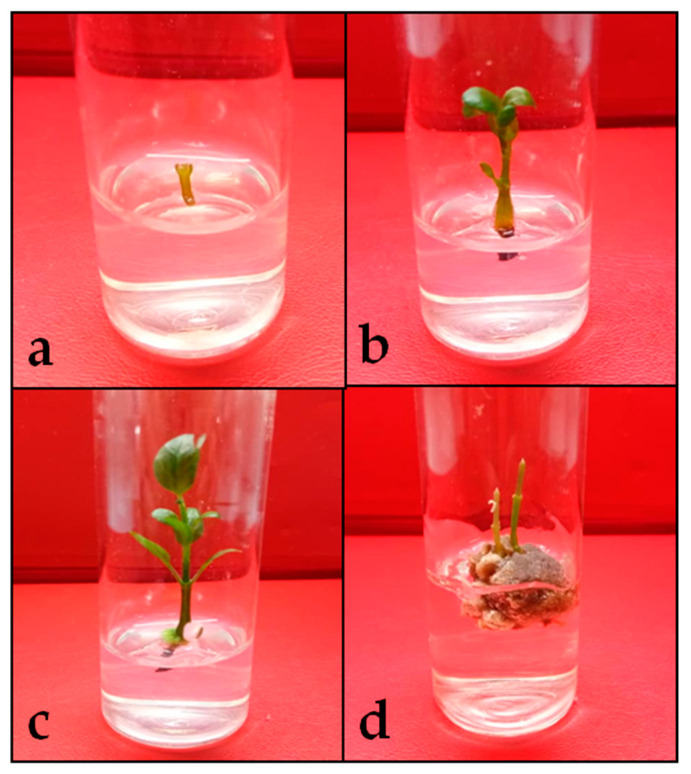
(**a**) The nodal segment of *C. nutans* that was used in the experiments: (**b**) the regenerated *C. nutans* at 4-week-old inoculated onto the Murashige and Skoog (MS) media supplemented with 12 µM BAP; (**c**) the *C. nutans* plantlet at 6-week-old inoculated onto the MS media supplemented with 12 µM BAP; (**d**) callus formation at the basal of *C. nutans* explant in the MS media supplemented with auxins.

The number and length of shoots of *C. nutans* were significantly affected by the inclusion of various concentrations of cytokinins in the culture medium. The highest number of shoots was produced in treatment with 8 µM kinetin, with a total of 1.57 shoots. The longest shoot was recorded from a medium supplemented with 12 µM BAP, with a length of 2.29 cm. Compared with all other treatments, only the treatment with 12 µM BAP was able to produce a shoot length of more than 2.00 cm. In the measured parameters, MS0 produced the lowest number of shoots (1.00 shoot) and the shortest shoot length (1.41 cm). In terms of yield production, the highest number and fresh weight of leaves were recorded from a treatment with 12 µM BAP yielding 4.78 leaves and 16.00 mg, respectively (Figure 1b). The fresh weight of leaves for the MS basal medium supplemented with various other concentrations of BAP increased gradually until the 12 µM BAP treatment. The fresh weight of leaves decreased two-fold in the 16 µM BAP concentration. A similar trend was also observed for the MS basal medium supplemented with various concentrations of kinetin. The fresh weight of leaves gradually increased until it reached a peak at 12 µM kinetin before dropping off.

#### 2.1.2. Synergistic Effect of Cytokinin and Auxins

In the present study, a treatment with 12 µM BAP was established as the best cytokinin concentration for the shoot induction of *C. nutans* nodal explants. The concentration was used with various concentrations of auxins. It is known that cytokinin is a plant hormone applied exogenously in basal media for the promotion of cell division and cell differentiation, while auxin is used to promote the cell elongation of explants.

Table 2 presents the effect of the supplementation of auxins in the basal medium. The highest shoot regeneration percentage (100%) for the parameter was obtained from the MS basal medium supplemented with 12 µM BAP without the addition of auxin. The lowest shoot regeneration percentage (44.44%) was recorded from treatment supplemented with auxin at 8 µM indole-3-butyric acid (IBA). The results implied that the addition of auxin significantly reduced the number of shoots and leaves being formed. The maximum numbers and length of shoots were recorded from control treatment with 1.67 shoots and 3.23 cm, respectively (Figure 1c). In the basal medium supplemented with auxin, 4 µM IBA recorded the maximum number and length of shoots with 1.44 shoots and 1.76 cm, respectively.

Synergistic effects between cytokinins and auxins also affected the number of leaves and fresh weight of leaves. The control treatment recorded the highest number of leaves (7.34 leaves) and fresh weight of leaves (21.57 mg), while the lowest was recorded from the treatment medium supplemented with 8 µM IBA with 0.89 leaves and 1.33 mg fresh weight of leaves. The addition of auxins in the basal medium of culture is known to be widely used for the induction of either the roots or callus. In the present study, the formation of callus was observed in all treatments including the control (Figure 1d). The treatment with 8 µM IBA accumulated the highest amount of callus with 69.00 mg. The lowest amount of callus was observed in the control treatment with 10.00 mg, implying that endogenous auxin had stimulated callus formation. Hence, the addition of exogenous auxin in the basal medium facilitated the formation of callus. The formation of callus at the base of the explants could have absorbed nutrients from the medium causing an interruption of nutrient absorption and stunting the growth of explants. In addition, root induction was also inhibited due to an excessive amount of callus formation.

#### 2.1.3. Optimization of Basal Medium Strength

In optimizing the strength of the basal medium in the in vitro propagation of *C. nutans* nodal explants, half-, full- and double-strength basal media were observed to vary in terms of the shoot regeneration responses. Table 3 presents the shoot regeneration capabilities of explants where the half-strength basal medium fared poorly (44.44%) compared with the full- and double-strength media which both achieved 100% shoot regeneration. Results on the number of shoots showed no significant difference between the full- and double-strength basal media. The full-strength basal medium recorded 1.67 shoots, while the double-strength basal medium recorded 1.44 shoots. The lowest number of shoots was recorded in half-strength basal medium, with a total of 0.67 shoots. On the other hand, the present study recorded a significant difference between the strength of the basal medium on the length of shoots. The longest shoot was obtained from the full-strength basal medium with 3.23 cm, followed by double-strength with 2.83 cm and 1.35 cm from the half-strength basal medium. The strength of the basal medium was also found to have a significant effect on the number and fresh weight of leaves produced by the explants. The highest number and fresh weight of leaves were produced from the full-strength basal medium with 7.33 leaves and 21.57 mg, respectively. Meanwhile, the half-strength basal medium produced the lowest number of leaves (3.00 leaves) and fresh weight of leaves (1.78 mg). The observations suggest that the number of leaves produced from each treatment had a significant effect on the fresh weight of leaves. There was no significant difference between the full- and double-strength of the basal medium on the number of leaves. However, the full-strength basal medium significantly produced a higher fresh weight of leaves. One possible reason for this phenomenon could be that double-strength basal medium produced a smaller leaf size compared to full-strength medium despite having a higher number of leaves. The half-strength basal medium also produced smaller leaves compared to the full-strength basal medium. The difference in terms of the fresh weight of leaves was also observed between basal medium strengths.

#### 2.1.4. Effects of Sucrose Concentrations on the Growth of *C*. *nutans*

One of the most important elements in plant tissue culture is carbon, as carbon plays a vital role as a source of energy and it controls the osmotic pressure of basal media. Sucrose is one of the most commonly used carbon sources in plant tissue culture due to its availability and cheaper price than other sugars. In the present experiment, the full-strength basal medium was supplemented with 12 µM BAP and various concentrations of sucrose (Table 4).

Based on the results in Table 4, the explants were able to achieve 100% shoot regeneration for sucrose concentrations of 20 to 35 g/L. A sucrose concentration higher than 35 g/L resulted in a decrease in the percentage of shoot regeneration. The lowest shoot regeneration was obtained from the highest sucrose concentration (50 g/L) with 33.33% regeneration. The highest number of shoots was recorded from sucrose concentrations of 30 and 35 g/L (1.67 shoots). However, the concentration of 30 g/L significantly produced the longest shoot (3.23 cm). The maximum number of leaves (8.0 leaves) was obtained from a treatment with 25 g/L sucrose. However, the number of leaves produced from the treatment was not significantly different from the treatment at 30 g/L (7.33 leaves). The highest number of leaves did not reflect the fresh weight of leaves obtained. The highest fresh weight of leaves was recorded from the treatment of 30 g/L sucrose, with a weight of 21.67 mg. Meanwhile, sucrose at a concentration of 25 g/L recorded a fresh weight of 14.67 mg. Although the sucrose concentration of 25 g/L recorded the highest number of leaves, the leaves produced were smaller than that of the sucrose concentration at 30 g/L. Among all the sucrose concentrations tested, the concentration of 50 g/L was least preferred in terms of the percentage of shoot regeneration (33.33%), number of shoots (0.33 shoots), length of shoots (1.18 cm), number of leaves (0.90 leaves) and fresh weight of leaves (0.68 mg).

### 2.2. Quantification of Phenolics Contents and Antioxidant Activities of Clinacanthus nutans

#### 2.2.1. Total Polyphenols, Phenolic Acids and Flavonoids Contents

The total polyphenols, total phenolic acids and total flavonoids contents in *C. nutans* leaves extracted aqueously at temperatures of 25 and 100 °C were evaluated and the results are presented in Table 5. The results show that at an aqueous temperature of 100 °C, cultured tissue and conventionally propagated leaves recorded higher total polyphenols, phenolic acids and flavonoids contents than at an aqueous temperature of 25 °C.

The highest total polyphenols content was recorded in tissue-cultured leaves extracted at 100 °C, with 2.77 mg GAE/g DW followed by the tissue-cultured leaves extracted at 25 °C with 2.15 mg GAE/g DW. For conventionally propagated leaves, there was no significant difference between the aqueous temperatures on the total polyphenols content of *C. nutans* leaves. An aqueous temperature of 25 and 100 °C exhibited 1.54 and 1.55 mg GAE/g DW of total polyphenols content, respectively.

The total phenolic acids content recorded ranged between 2.12 and 4.29 mg GAE/g DW. The highest total phenolic acids content was produced by an extraction of tissue-cultured leaves at 100 °C with 4.29 mg GAE/g DW. Meanwhile, the lowest total phenolic acids content was recorded from the treatment at 25 °C conventionally propagated leaves extract with 2.12 mg GAE/g DW.

The total flavonoids content recorded in the tissue-cultured leaves was significantly higher than conventionally propagated leaves. The highest total flavonoids content exhibited by the extraction of tissue-cultured leaves at 100 °C was 21.76 mg RE/g DW followed by the tissue-cultured leaves extracted at 25 °C with 9.10 mg RE/g DW. The lowest total flavonoids content was recorded by conventionally propagated leaves extracted at 25 °C with 5.26 mg RE/g DW, which was four times lower than the highest total flavonoids content recorded.

#### 2.2.2. Antioxidant Activities

The antioxidant activities of *C. nutans* leaf extracts, propagated via conventional and tissue culture techniques, were measured using five in vitro antioxidant assays including 2,2-diphenyl-1-picryl-hydrazyl-hydrate (DPPH) free radical scavenging activity, 2,2-azino-bis(3-ethylbenzothiazoline-6-sulfonic acid) (ABTS) scavenging activity, ferric reducing antioxidant power (FRAP), superoxide anion radical scavenging activity and iron (II) chelating activity. All antioxidant assays were measured using a UV-Vis spectrophotometer at a specific absorbance. A standard graph was generated using trolox as a standard for the DPPH free radical scavenging activity (y = 0.0007x + 0.0865, R2 = 0.989), ABTS scavenging activity (y = 0.0106x + 0.09, R2 = 0.997) and ferric reducing antioxidant power (y = 0.0044x + 0.066, R2 = 0.999). Based on the results presented in Table 6, the DPPH free radical scavenging activity recorded for the aqueously extracted tissue-cultured and conventionally propagated leaves of *C. nutans* ranged between 2.44 and 3.20 mg TE/g DW. The highest DPPH free radical scavenging activity was recorded from the aqueously extracted tissue-cultured leaves at 100 °C with 3.20 mg TE/g DW, followed by the aqueously extracted tissue-cultured leaves at 25 °C with 2.98 mg TE/g DW. The lowest DPPH free radical scavenging activity was recorded for the aqueously extracted conventionally propagated leaves at 100 °C with 2.44 mg TE/g DW.

The measurement of the ABTS scavenging activity is based on the ability of an extract to scavenge the ABTS cation radical. The reaction between the ABTS cation radical and extract reduces the dark blue color of ABTS. The results obtained in the present study showed that the aqueously extracted tissue-cultured leaves at 100 °C significantly exhibited the highest ABTS scavenging activity with 1.50 mg TE/g DW (Table 6). There was no significant difference between the aqueously extracted tissue-cultured leaves at 25 °C, aqueously extracted conventionally propagated leaves at 25 °C and 100 °C with 1.48, 1.47 and 1.46 mg TE/g DW of ABTS scavenging activity, respectively.

Based on Table 6, the FRAP value recorded in the tissue-cultured leaf extract was significantly higher than the conventionally propagated leaf extract. The FRAP value recorded was significantly affected by both factors (sources of leaves and aqueous temperature). The highest FRAP value from tissue-cultured leaves extracted at 100 °C was 9.76 mg TE/g DW. The second highest FRAP value from tissue-cultured leaves extracted at 25 °C was 7.13 mg TE/g DW. The conventionally propagated leaves extracted at 100 °C recorded a higher FRAP value with 5.31 mg TE/g DW compared to the 25 °C extraction, which had a value of 4.27 mg TE/g DW.

The superoxide anion radical scavenging was conducted to study the ability of plant samples to scavenge superoxide anion radicals. The data revealed that the sources of leaves and aqueous temperatures significantly affected the superoxide anion radical scavenging activity. The highest superoxide anion inhibition percentage was recorded from the tissue-cultured leaves extracted at 100 °C with 53.74% inhibition, followed by the tissue-cultured leaves extracted at 25 °C with 52.22% inhibition (Table 7). The lowest superoxide anion inhibition percentage was recorded from the conventionally propagated leaves extracted at 25 °C with 39.84% inhibition.

Antioxidants, besides acting as scavengers of free radicals, also act as chelators of metal ions such as copper (II) and iron (II). In contrast with other antioxidant activities, the highest was recorded from the tissue-cultured leaf extracts. The highest iron (II) chelating activity was produced from the conventionally propagated leaf extracts. Based on the results in Table 7, the highest percentage for the iron (II) chelating activity was significantly exhibited from the conventionally propagated leaves extracted at 25 °C with 69.24% inhibition. Meanwhile, there was no significant difference between the tissue-cultured leaves extracted at 25 and 100 °C and the conventionally propagated leaves extracted at 100 °C, with the iron (II) chelating activity recorded at 54.33, 53.50 and 51.33% inhibition, respectively.

#### 2.2.3. Correlation Analysis between Variables

The correlation between the phenolics content (total polyphenols, phenolic acids and flavonoids contents) and antioxidant activities, including the DPPH free radical scavenging activity, FRAP assay, ABTS scavenging activity, superoxide anion radical scavenging activity and iron (II) chelating activity, were evaluated using Pearson’s correlation analysis (Table 8).

The results show that all variables, except the iron (II) chelating activity, were positively correlated. The variables for the total polyphenols, phenolic acids, flavonoids, DPPH free radical scavenging activity, FRAP assay and ABTS scavenging activity exhibited a high correlation between the variables measured with *P* > 0.70. For the superoxide anion radical scavenging activity, a significant correlation was recorded against all variables except the DPPH free radical scavenging activity and ABTS scavenging activity. Meanwhile, the iron (II) chelating activity showed a nonsignificant correlation against all variables except the superoxide anion radical scavenging activity. Pearson’s correlation analyses confirmed that the phenolics contents in the *C. nutans* leaf extracts acted as good scavenging agents but not as a chelating agents.

## 3. Discussion

In this present study, the optimum basal medium formulation by the alteration of plant growth regulators, basal medium strength and sucrose concentration for the in vitro propagation of *C. nutans* were established. In plant tissue culture, plant growth regulators are one of the most important factors affecting the growth of explants. In a single node culture of *C. nutans*, the shoot induction was affected by the application of cytokinin in the culture medium. The synthesis of cytokinin in plants becomes limited due to injury when plant parts are excised into smaller sizes [15,16]. Hence, cytokinin was supplemented in the basal medium to support shoot induction, cell division and cell differentiation. In the present study, 12 µM BAP was more favorable than other cytokinins in the initial stage of the shoot induction of *C. nutans*. A study on *Justicia gendarussa* (Acanthaceae) showed that the supplementation of BAP in an MS basal medium exhibited the highest percentage of shoot induction, number of shoots and shoot length, compared to a basal medium supplemented with kinetin and thidiazuron (TDZ) [17,18]. In addition, similar studies on *Andrographis paniculate*, which belong to the same family as *C. nutans* and *J. gendarussa*, also recorded that the application of BAP was able to produce a higher percentage of shoot regeneration and number of shoots [19].

The integration of cytokinin and auxin is the most commonly used technique in plant tissue cultures to increase the multiplication and proliferation rates of plantlets. However, the present study observed that the application of auxin significantly reduced the percentage of shoot regeneration, number of shoots, length of shoots, number of leaves and fresh weight of leaves. A supplementation with auxin resulted in the production of a massive amount of callus. Auxin is supplemented in plant tissue culture basal medium to facilitate cell elongation and induce rooting. In several cases, due to the presence of endogenous auxin in plants, callus is formed [20]. The current findings on the effects of auxin were also supported by a previous study on *Tylophora indica* in which a combination of BAP and IBA in as low as 1.23 µM of a basal medium significantly reduced the growth of the plant compared to same cultured in BAP alone [21]. Similarly, another study on *Spilanthes acmella* Murr. reported that the supplementation of cytokinin alone in the basal medium produced better growth [22]. In both of these studies, the callus formation was observed in the basal regions of the explants [21,22].

The effect of the basal medium strength on the in vitro growth of *C. nutans* was also investigated. The full-strength MS basal medium yielded superior results for all the parameters studied. Similar results were also reported in a previous study by Chen et al. [23], where a full-strength MS basal medium supplemented with 4.44 µM BAP and 0.11 µM NAA produced the highest multiplication rate and the longest shoots for *C. nutans*. The study reported that as the basal medium strength was reduced to half, the number of leaves and length of shoots significantly decreased. Besides, the growth rate slowed and the explants stunted. Based on the growth vigor and morphological changes, the full-strength basal medium produced more vigorous shoots and greener leaves compared to half-strength basal medium which produced yellowish leaves. The full-strength basal medium was also found to be most effective for the micropropagation of *Ocimum basilicum* [24], *Bacopa monnieri* [25] and *Thymus hyemalis* [26]. All of these findings were in agreement with current findings, indicating the effectiveness of the full-strength MS basal medium for the medicinal plant studied. On the contrary, the double-strength MS basal medium appeared to be a toxic basal medium, while quarter- and half-strength MS basal media were found to cause nutrient deficiencies in explants [24]. The MS basal medium is known to be a balanced culture medium in terms of macronutrients, micronutrients and vitamins suitable for a wide range of plant species.

A continuous supply of carbon is important in plant tissue cultures in order to support plant growth. Several studies have been conducted on different species of plants. In a study by Naz et al. [27], carbon sources including sucrose, glucose and fructose at concentrations from 10 to 50 g/L were supplemented in the basal medium of *Althaea officinalis*. The results indicated that 30 g/L of sucrose exhibited the best result for all the parameters measured. Similarly, Reddy et al. [28] reported similar results for *Ceropegia ensifolia*. The addition of 30 g/L sucrose was found to be the optimum for the growth of *Thymus hyemalis* compared to other types of carbon sources including glucose, fructose, mannitol and sorbitol [17]. The studies implied that a sucrose concentration higher than 30 g/L results in a decrease in the multiplication and growth rate of plants [24,25,26,27,28,29,30,31,32,33,34,35,36,37,38]. These findings were in agreement with the present study in which 30 g/L of sucrose was established as the optimum for the growth of *C. nutans*. The literature has previously found that sucrose is found to be the most suitable carbon source for in vitro propagation compared to glucose, fructose and other types of sugar, and is the most common carbohydrate present in the phloem saps of plants. Hence, the translocation process in the plant will be eased [29,30]. The addition of sucrose at more than the optimum level would increase the osmotic pressure of the basal medium, causing the water and mineral uptake by explants to be inhibited. Gas exchange in culture vessels would be limited resulting in an increased toxicity of the basal medium [31].

In the present study, the propagation technique (tissue culture and conventional propagation) and the effects of aqueous temperatures (25 and 100 °C) on the extraction of phenolics contents and antioxidant activities were studied. Based on the total polyphenols, total phenolic acids and total flavonoids contents measured, tissue-cultured leaves significantly produced higher values on all the parameters under study compared to the conventionally propagated leaves. The extractions at 100 °C recorded a higher amount of total polyphenols, total phenolic acids and total flavonoids from both sources of leaves compared to extractions at 25 °C. Pandey et al. [32] conducted a study on *Origanum vulgare*, comparing the leaves of the tissue-cultured plant and conventionally propagated mother plants. They found that the leaves of tissue-cultured plants significantly produced higher total phenolics, flavonoids and tannins contents than the leaves of the mother plant. In another study comparing the field-grown and tissue-cultured plants of *Lavandula angustifolia* var., Ellagance Blue, Blue River and Munstead found that tissue-cultured plants of all three varieties exhibited a higher total polyphenols content compared to field-grown plants [33]. A study by Bose et al. [34] reported a similar finding on mother and tissue-cultured plants of *Nardostachys jatamansi* extracted with different extraction solvents including methanol, acetone, chloroform, acetonitrile and water. Their results showed that the leaves of tissue-cultured plants significantly produced higher total phenolics, flavonoids, alkaloids and tannins contents in all the extraction solvents tested. The variations in phenolics content could be due to different extraction processes such as different extraction temperatures and different sources of plants. The concentration of secondary metabolites in plants could be affected by hormonal contents as well as endogenous physiological changes in the plants. According to Umebese and Falana [35], the accumulation of the phenolics content is generally low when plants are grown under nonstress conditions. The accumulation of phenolics increases when plants are exposed to stress conditions.

In an antioxidant activities study, it was observed that leaves from tissue-cultured plants exhibited higher DPPH, ABTS and FRAP values compared to leaves from conventionally propagated plants. Studies by Amoo et al. [36] and Bhattacharyya et al. [37] on *Aloe arborescene* and *Dendrobium thyrsiflorum*, reported similar findings. The strong antioxidant activities in plant extracts could be due to the presence of high concentrations of secondary metabolites such as phenolics, tannins and alkaloids contents. High concentrations of secondary metabolites in plant samples enhance their redox properties and the ability to scavenge free radicals [38,39].

A study on superoxide anion radical scavenging activities showed that leaves from tissue-cultured plants produced a higher inhibition percentage in comparison with leaves from conventionally propagated plants. Similar findings were reported on shoot and root extracts of tissue-cultured *Salvia miltiorrhiza*, producing a higher inhibition percentage of superoxide anion radical scavenging activity compared to extracts from seed-derived plants [40]. Superoxide radicals are known to be extremely harmful as they can act as a precursor for the production of reactive oxygen species. An excessive amount of superoxide anions produced in cells increases dismutation and leads to hydrogen peroxide production and thereby increases the oxidative stress levels in the human body [41]. Hence, the ability of plant samples to scavenge superoxide anion is important to reduce the concentration of superoxide radicals and lower oxidative stress levels.

The results in the present study on the phenolics content and antioxidant activities recorded that aqueous temperature of 100 °C was more prominent in producing a higher phenolic content and antioxidant activities compared to the aqueous temperature of 25 °C. The results showed that the coefficient of diffusion and phenolic contents solubility increased as the aqueous temperature was increased. An increased solubility of phenolic contents by a higher temperature favors the release of bound phenolics in samples with cellular constituents of the plant cell breakdown which leads to an increase in the cell membrane permeability [41]. In addition, an elevated extraction temperature causes the rate of thermally stable antioxidants to be higher than the rate of decomposition of less soluble antioxidants [42]. This was proven by 100 °C aqueous extraction of *C. nutans* leaves to gain higher antioxidant activities.

## 4. Materials and Methods

### 4.1. In Vitro Propagation of Clinacanthus nutans via Single Node Culture

#### 4.1.1. Planting Materials and Sterilization

The mother plants of *C. nutans* used in this study were purchased from the local nursery located at Kuala Pilah, Negeri Sembilan, Malaysia. Prior to experimentation, the plants were placed and maintained under natural environmental conditions. The *C. nutans* used in this study were deposited at the herbarium of the Biodiversity Unit, Institute of Bioscience, Universiti Putra Malaysia with a voucher number of MFI 0015/18.

The sterilization process was carried out by collecting the nodal segments of *C. nutans* from the healthy mother plants. The explants were placed under running tap water with an additional commercial detergent for 30 min to remove the soil debris and contaminants. Further sterilization of explants was conducted with 50% commercial bleach (5.25% sodium hypochlorite) and shaken for 20 min. The explants were rinsed three times with sterile distilled water to remove all the disinfectants. Finally, the aseptic explants were excised into approximate sizes of 1 ± 0.5 cm for the experiments (Figure 1a).

#### 4.1.2. Culture Medium and Conditions

The basal medium was prepared using the formation described by Murashige and Skoog [14] which is referred to as MS basal medium. The basal medium was supplemented with 30 g/L sucrose and 3.0 g/L Gelrite as a gelling agent with the pH adjusted to 5.75 prior to the autoclave. The basal medium was autoclaved for 20 min at 121 °C at a pressure of 1.05 kg cm^−2^.

#### 4.1.3. Shoot Induction of *C. nutans* as Affected by Cytokinins

The shoot induction of *C. nutans* was performed by supplementing the MS basal media with different types and concentrations of cytokinins including 6-benzylaminopurine (BAP) and 6-furfurlaminopurine (kinetin) at 4, 8, 12 and 16 µM concentrations. The MS basal media without cytokinin served as the control. The data on the shoot regeneration percentage, number of shoots, length of shoots, number of leaves and fresh weight of leaves were recorded after an incubation of four weeks.

#### 4.1.4. Synergistic Effect of Cytokinin and Auxins

The MS basal media was fortified with the best cytokinin from the previous experiment. In addition, the different types and concentrations of auxins were supplemented into the basal media. The auxins used were indole-3-butyric acid (IBA) and 1-napthaleneacetic acid (NAA) at the concentrations of 2, 4, 6 and 8 µM. The MS basal media without auxin supplementation served as the control. The data on the shoot regeneration percentage, number of shoots, length of shoots, number of leaves, fresh weight of leaves and callus formation (if any) were recorded after six weeks of the incubation period.

#### 4.1.5. Optimization of Basal Medium Strength

As for the optimization of basal medium strength, the MS basal medium was modified into half-, full- and double-strengths. The full-strength MS basal medium was used as a control. The data on the shoot regeneration percentage, number of shoots, length of shoots, number of leaves and fresh weight of leaves were recorded after six weeks of incubation.

#### 4.1.6. Effect of Sucrose Concentrations on the Growth of *C*. *nutans*

In order to test the effects of sucrose on growth, sucrose at concentrations of 20, 25, 30, 35, 40, 45 and 50 g/L were fortified into the MS basal medium. The sucrose concentration of 30 g/L served as the control for this experiment. The data on the shoot regeneration percentage, number of shoots, length of shoots, number of leaves and fresh weight of leaves were recorded at the end of a six-week incubation period.

#### 4.1.7. Experimental Unit and Culture Maintenance

All the experiments were conducted with three replications and six explants per replication. The explant was inoculated individually in a vial 7.5 cm in height (approximately 7 ± 1 mL basal medium). All the cultures were incubated in a culture room maintained at a temperature of 25 °C under 16 h of light and 8 h of dark using white fluorescence light irradiation of 2000 lux.

### 4.2. Quantification of Phenolics Contents and Antioxidant Activities of Clinacanthus nutans

#### 4.2.1. Planting Materials

The *C. nutans* plant was propagated using two techniques which were plant tissue culture and conventional propagation (stem cutting) techniques. The leaves were collected from six-week-old tissue-cultured and conventionally propagated *C*. *nutans*. The tissue-cultured plant of *C. nutans* was propagated on the best MS basal medium formulation obtained in Section 2. Meanwhile, a conventionally propagated plant was maintained under full sunlight and organic fertilizer was applied every five weeks.

Before the experiment, the leaves were cleaned to remove the contaminant, soil and basal media that attached to it. The leaves were dried in an oven at a temperature of 55 °C for 48 h or until the weight remained constant. The leaves were kept in an airtight container until the antioxidant study was carried out.

#### 4.2.2. Chemicals and Reagents

Folin-Ciocalteu reagent, sodium carbonate, sodium nitrite, aluminium chloride, sodium hydroxide, gallic acid, rutin, 2,2-diphenyl-1-picrylhydrazyl (DPPH), methanol, 6-hydroxy-2,5,7,8-tetramethylchroman-2-carboxylic acid (Trolox), acetate buffer, 2,4,6-tri (2-pyridyl)-s-triazine (TPTZ), hydrochloric acid, iron (lll) chloride hexahydrate, nitro blue tetrazolium (NBT), nicotinamide adenine dinucleotide (NADH), Tris-HCl buffer, phenazine methosulphate (PMS), ferrous chloride, ferrozine, 2,2′-azino-bis(3-ethylbenzothiazoline-6-sulphonic acid) (ABTS) and potassium persulphate were used. All the chemicals and reagents were of analytical grade.

#### 4.2.3. Preparation of Extract

The extraction was carried out according to the method explained by Hakiman and Maziah [43] with minor modifications. Briefly, 0.25 g of dried leaves of *C. nutans* was ground using a commercial blender and placed in the 50 mL vials, and then covered with aluminum foil. Then, 12.5 mL of aqueous at a temperature of 25 °C was added. The vials were placed on an orbital shaker for an hour in the dark room at room temperature. The samples were filtered using filter paper No. 1 and the extracts were used for antioxidant analysis. The procedure was repeated and the samples were extracted with aqueous at a temperature of 100 °C.

#### 4.2.4. Total Polyphenols Content

The total polyphenols content was determined according to the method explained by Marinova et al. [44]. In each vial, 50 µL extract and 1.25 mL of Folin-Ciocalteu reagent, that was diluted 10 times, were added. The mixture was incubated for 5 min and 1.25 mL of sodium carbonate was added. The reaction mixture was incubated for an hour at room temperature. The absorbance was measured at 725 nm using a spectrophotometer. The total polyphenols content in the extract was calculated by a constructed standard curve of absorbance against different concentrations of gallic acid. The total polyphenols content of the extract was expressed as mg gallic acid equivalent per gram dry weight of the sample (mg GAE/g DW).

#### 4.2.5. Total Phenolic Acids Content

The total phenolic acid content was conducted according to the method described by Singleton and Rossi [45]. Briefly, 0.5 mL of extract, 4.5 mL of distilled water and 0.5 mL of Folin-Ciocalteu reagent were added and mixed thoroughly using a vortex machine. After 5 min, 5 mL of 7% sodium carbonate was added. The final volume of the reaction mixture was adjusted to 12.5 mL by the addition of 2 mL of distilled water. The reaction mixture was incubated for 90 min at room temperature and the absorbance was measured at 750 nm. The total phenolic acids content was calculated by a constructed standard curve of absorbance against different concentrations of gallic acid. The total phenolic acid content was expressed as mg gallic acid equivalent per gram dry weight of the sample (mg GAE/g DW).

#### 4.2.6. Total Flavonoids Content

The total flavonoids content was determined using an aluminum chloride colorimetric method explained by Marinova et al. [44]. A total of 0.5 mL of extract was added into a test tube that contained 2 mL of distilled water. Then, 150 µL of 5% sodium nitrite was added and the mixture was incubated for 5 min. After the incubation, 150 µL of 10% aluminum chloride was added to the mixture. At the sixth minute, 1 mL of 1 M sodium hydroxide and 1.2 mL of distilled water were added and the mixture was incubated for 90 min at room temperature. The absorbance was measured using a spectrophotometer at 510 nm. The standard curve of absorbance against different concentrations of rutin was constructed and the total flavonoids content of the extract was expressed as mg rutin equivalent per gram dry weight (mg RE/g DW).

#### 4.2.7. DPPH Free Radical Scavenging Activity

DPPH free radical scavenging was conducted according to the method employed by Wong et al. [46]. Prior to the experiment, DPPH was prepared in methanol with a concentration 0.1 mM and the initial absorbance of methanolic DPPH was measured immediately at an absorbance of 515 nm. A total of 20 µL extract was added into 1.5 mL of 0.1 mM of the methanolic DPPH solution. The mixture was incubated at room temperature for 30 min and the absorbance was measured at 515 nm. The standard curve of absorbance against different concentrations of trolox was constructed and the DPPH value was expressed as mg trolox equivalent per gram dry weight (mg TE/g DW).

#### 4.2.8. ABTS Scavenging Activity

The ABTS scavenging activity was conducted according to the method by Re et al. [47]. Prior to the experiment, 7 mM of ABTS stock was mixed with 2.45 mM potassium persulphate with a ratio of 1:1 and incubated for 16 h in the dark at room temperature. After the incubation, the ABTS+ solution was diluted with methanol and the absorbance was adjusted to 0.700 ± 0.05 at 734 nm. Then, 0.1 mL of extract was mixed with 0.9 mL of diluted ABTS+. The reaction mixture was incubated for 15 min at room temperature and the absorbance was taken at 734 nm. The standard curve of absorbance against different concentrations of trolox was constructed and the ABTS value was expressed as mg trolox equivalent per gram dry weight (mg TE/g DW).

#### 4.2.9. Ferric Reducing Antioxidant Power (FRAP)

The FRAP assay was conducted to measure the reduction in Fe^3+^ to Fe^2+^ under an acidic condition. The FRAP reagent was prepared prior to experiment by a mixed 0.3 M acetate buffer (pH 3.6), 20 mM FeCl_3_,6H_2_O and 10 mM 2,4,6 tri(2-pyridyl)-s-triazine (TPTZ) in 40 mM HCL at a ratio of 10:1:1, respectively. Then, 100 µL of the extract was added into a vial that contained 1.5 mL of FRAP reagent. The reaction mixture was incubated for 30 min in the water bath at a temperature of 37 °C. The reaction mixture was cooled down and the absorbance was measured at 593 nm. The standard curve of absorbance against different concentrations of trolox was constructed and FRAP value was expressed as mg trolox equivalent per gram dry weight (mg TE/g DW) [48].

#### 4.2.10. Superoxide Anion Radical Scavenging Activity

The superoxide anion radical scavenging activity is based on the reduction in nitro blue tetrazolium (NBT) in the presence of nicotamide adenine dinucleotide (NADH) and phenazine methosulphate (PMS). Briefly, 0.25 mL of 0.3 mM of NBT, 0.25 mL of 0.936 mM of NADH, 0.5 mL of extract and 0.5 mL of 16 mM Tris-HCl buffer (pH 8.0) were mixed. The reaction was initiated by adding 0.25 mL of 0.12 mM PMS and incubated for 5 min at a temperature of 25 °C. The control was prepared without the addition of extract. The absorbance was measured at 560 nm and the percentage of inhibition was calculated by Inhibition (%) = (1 − A560 Sample/A560 Control) × 100 [49].

#### 4.2.11. Iron (II) Chelating Activity

The iron (II) chelating activity was carried out according to the method explained by Prieto et al. [50]. Briefly, 400 µL of the extract was mixed with 50 µL of 2 mM ferrous chloride. The reaction mixture was initiated by adding 200 µL of 5 mM of ferrozine and incubated for 10 min at room temperature. The absorbance was measured at 562 nm and the percentage of the iron (II) chelating activity was calculated by Chelating (%) = (1 − A562 Sample/A562 Control) × 100.

### 4.3. Statistical Analysis

All the experiments were laid out in a completely randomized design (CRD) with three replications. Data were analyzed using analysis of variance (ANOVA) to compare the significant differences between treatments using Statistical Analysis Software (SAS ver. 9.4). The comparison of means was conducted using Duncan’s Multiple Range Test (DMRT) at *P* = 0.05. The correlation between variables was conducted using a Pearson’s correlation analysis with indicator *P* < 0.30 indicating a negligible correlation, *P* < 0.50 indicating a low correlation, *P* < 0.70 indicating a moderate correlation, *P* < 0.90 indicating a high correlation and *P* < 1.0 indicating a very high correlation.

## 5. Conclusions

In the present study, different plant growth factors, including plant growth regulators, basal medium strength and sucrose concentration, were used to determine the most suitable formulation for a plant tissue culture of *C. nutans*. The study reported that the full-strength MS basal medium supplemented with 12 µM BAP and 30 g/L sucrose exhibited the best result in terms of a high percentage of shoot regeneration, number of shoots, length of shoots, number of leaves and fresh weight of leaves. In the next experiment, the phenolics content and antioxidant activities were quantified using 25 and 100 °C temperatures. The study found that tissue-cultured leaves extracted at 100 °C was more prominent by exhibiting higher total polyphenols (2.77 mg GAE/g DW), total phenolic acids (4.29 mg GAE/g DW), total flavonoids (21.76 mg RE/g DW), DPPH free radical scavenging activity (3.20 mg TE/g DW), ABTS scavenging activity (1.50 mg TE/g DW), FRAP assay (9.76 mg TE/g DW) and superoxide anion radical scavenging activity (53.74% inhibition). Meanwhile, the highest iron (II) chelating activity was recorded from conventionally propagated leaves extracted at 25 °C with 69.24% inhibition. Based on the results obtained, 100 °C was found to be the most suitable extraction temperature for the dried tissue-cultured leaves of *C. nutans*. This study successfully established a protocol for the propagation of *C. nutans* via a plant tissue culture study. Besides that, this present study also proved that tissue-cultured plants were able to produce high phenolic contents and antioxidant activities. Hence, these findings may contribute to the production of raw materials of *C. nutans* to fulfill the demand in the pharmaceutical industry.

## Figures and Tables

**Table 1:** The effect of cytokinins on the shoot regeneration percentage, number of shoots, length of shoots, number of leaves and leaves fresh weight of nodal segments of *C*. *nutans*.

Treatment	Shoot Regeneration (%)	Number of Shoots	Length of Shoots (cm)	Number of Leaves	Leaves Fresh Weight (mg)
MS0	88.89 b	1.00 e	1.41 d	2.22 d	4.00 e
4 µM BAP	88.89 b	1.30 c	1.95 b	3.44 c	8.67 bc
8 µM BAP	100 a	1.43 b	1.92 b	3.67 bc	9.00 bb
12 µM BAP	100 a	1.30 c	2.29 a	4.78 a	16.00 a
16 µM BAP	100 a	1.43 b	1.96 b	3.78 bc	8.67 bc
4 µM kinetin	88.89 b	1.10 d	1.60 c	2.11 d	4.00 e
8 µM kinetin	100 a	1.57 a	1.67 c	3.89 b	6.00 de
12 µM kinetin	100 a	1.30 c	1.92 b	3.44 c	7.33 bcd
16 µM kinetin	100 a	1.30 c	1.88 b	3.67 bc	6.33 cde

Data are the means of three replications. Means followed by the same letter within the columns are not significantly different at *P* = 0.05 using Duncan’s Multiple Range Test (DMRT) mean separation.

**Table 2:** The effect of cytokinin and auxins on the shoot regeneration percentage, number of shoots, length of shoots, number of leaves, leaves fresh weight and callus fresh weight of nodal segments of *C. nutans*.

Treatment (MS + 12 µM BAP + Auxin)	Shoot Regeneration (%)	Number of Shoots	Length of Shoots (cm)	Number of Leaves	Leaves Fresh Weight (mg)	Callus Fresh Weight (mg)
Control	100 a	1.67 a	3.23 a	7.34 a	21.57 a	10.00 d
2 µM IBA	77.78 c	1.11 bc	1.32 d	3.22 bc	3.43 bc	37.57 bcd
4 µM IBA	88.89 b	1.44 ab	1.76 b	4.66 b	6.90 b	42.23 abcd
6 µM IBA	66.67 d	0.78 cd	1.35 d	2.33 bc	3.53 bc	53.10 abc
8 µM IBA	44.44 f	0.44 e	1.03 f	0.89 c	1.33 c	69.00 ab
2 µM NAA	88.89 b	1.11 bc	1.59 c	3.44 bc	3.53 bc	15.13 cd
4 µM NAA	77.78 c	0.89 cd	1.27 de	1.67 c	2.10 c	81.43 a
6 µM NAA	77.78 c	0.89 cd	1.28 de	2.22 bc	1.77 c	63.53 ab
8 µM NAA	55.56 e	0.67 d	1.13 ef	1.67 c	2.23 c	60.53 ab

Data are the means of three replications. Means followed by the same letter within the columns are not significantly different at *P* = 0.05 using Duncan’s Multiple Range Test (DMRT) mean separation.

**Table 3:** The effect of basal medium strength on the shoot regeneration percentage, number of shoots, length of shoots, number of leaves and fresh weight of leaves of nodal segments of *C. nutans*.

MS Basal Medium Strength	Shoot Regeneration (%)	Number of Shoots	Length of Shoots (cm)	Number of Leaves	Leaves Fresh Weight (mg)
Half-strength	44.44 b	0.67 b	1.35 c	3.00 b	1.78 c
Full-strength	100 a	1.67 a	3.23 a	7.33 a	21.57 a
Double-strength	100 a	1.44 a	2.83 b	6.67 a	10.33 b

Data are the means of three replications. Means followed by the same letter within the columns are not significantly different at *P* = 0.05 using Duncan’s Multiple Range Test (DMRT) mean separation.

**Table 4:** The effect of sucrose concentrations on the shoot regeneration percentage, number of shoots, length of shoots, number of leaves and fresh weight of leaves of nodal segments of *C*. *nutans*.

Sucrose Concentration (g/L)	Shoot Regeneration (%)	Number of Shoots	Length of Shoots (cm)	Number of Leaves	Leaves Fresh Weight (mg)
20	100 a	1.33 b	2.71b	6.97 ab	16.67 b
25	100 a	1.56 ab	3.06 a	8.00 a	14.67 bc
30	100 a	1.67 a	3.23 a	7.33 a	21.57 a
35	100 a	1.67 a	2.78 b	6.23 b	11.67 c
40	77.78 b	1.00 c	2.39 c	4.57 c	6.00 d
45	66.67 c	0.78 d	1.70 d	2.80 d	5.00 d
50	33.33 d	0.33 e	1.18 e	0.90 e	0.68 e

Data are the means of three replications. Means followed by the same letter within the columns are not significantly different at *P* = 0.05 using Duncan’s Multiple Range Test (DMRT) mean separation.

**Table 5:** Total polyphenols, phenolic acids and flavonoids contents of tissue-cultured and conventionally propagated leaves of *C*. *nutans*.

Source of Leaves	Aqueous Temperature (°C)								
	Polyphenols (mg GAE/g DW)			Phenolic Acids (mg GAE/g DW)			Flavonoids (mg RE/g DW)		
	25	100	Mean	25	100	Mean	25	100	Mean
Tissue culture	2.15 b	2.77 a	2.46 A	3.13 b	4.29 a	3.71 A	9.10 b	21.76 a	15.43 A
Conventional	1.54 c	1.55 c	1.55 B	2.12 d	2.76 c	2.44 B	5.26 d	6.68 c	5.97 B
Mean	1.85 B	2.16 A		2.63 B	3.53 A		7.18 B	14.22 A	

Means followed by the same letter in the same columns and rows are not significantly different at *P* = 0.05 using Duncan’s Multiple Range Test.

**Table 6:** 2,2-diphenyl-1-picryl-hydrazyl-hydrate (DPPH) free radical scavenging activity, 2,2-azino-bis(3-ethylbenzothiazoline-6-sulfonic acid) (ABTS) scavenging activity and ferric reducing antioxidant power (FRAP) assay of tissue-cultured and conventionally propagated leaves of *C. nutans*.

Source of Leaves	Aqueous Temperature (°C)								
	DPPH (mg TE/g DW)			ABTS (mg TE/g DW)			FRAP (mg TE/g DW)		
	25	100	Mean	25	100	Mean	25	100	Mean
Tissue culture	2.98 b	3.20 a	3.10 A	1.48 b	1.50 a	1.49 A	7.13 b	9.76 a	8.45 A
Conventional	2.72 c	2.44 d	2.58 B	1.47 b	1.46 b	1.47 B	4.27 d	5.31 c	4.79 B
Mean	2.85 A	2.82 B		1.48 A	1.48 A		5.70 B	7.54 A	

Means followed by the same letter in the same columns and rows are not significantly different at *P* = 0.05 using Duncan’s Multiple Range Test.

**Table 7:** Superoxide anion radical scavenging activity and iron (II) chelating activity of tissue-cultured and conventionally propagated leaves of *C*. *nutans*.

Source of Leaves	Aqueous Temperature (°C)					
	Superoxide Inhibition (%)			Iron (II) Chelating Activity (%)		
	25	100	Mean	25	100	Mean
Tissue culture	52.22 b	53.74 a	52.98 A	54.33 b	53.50 b	53.92 B
Conventional	39.84 d	48.97 c	44.41 B	69.24 a	51.33 b	60.29 A
Mean	46.03 B	51.36 A		61.79 A	52.42 B	

Means followed by the same letter in the same columns and rows are not significantly different at *P* = 0.05 using Duncan’s Multiple Range Test.

**Table 8:** Pearson’s correlation analysis between variables.

Variable	TPP	TPC	TFC	DPPH	FRAP	ABTS	O_2_^−^	Fe^2+^
TPP	1							
TPC	0.95 **	1						
TFC	0.95 **	0.96 **	1					
DPPH	0.92 **	0.76 **	0.81 **	1				
FRAP	0.98 **	0.99 **	0.95 **	0.84 **	1			
ABTS	0.86 **	0.78 **	0.86 **	0.85 **	0.81 **	1		
O_2_^−^	0.76 **	0.86 **	0.69 **	0.53 ^ns^	0.85 **	0.46 ^ns^	1	
Fe^2+^	−0.39 ^ns^	−0.58 ^ns^	−0.38 ^ns^	−0.08 ^ns^	−0.52 ^ns^	−0.11 ^ns^	−0.82 **	1

Notes: **: Significant correlation at *P* < 0.05; ns: Non-significant correlation; TPP: Total polyphenols content; TPC: Total phenolic acids content; TFC: Total flavonoids content; DPPH: DPPH free radical scavenging activity; FRAP: Ferric reducing antioxidant power; ABTS: ABTS scavenging activity; O_2−_: -Superoxide anion radical scavenging activity and; Fe^2+^: Iron (II) chelating activity, respectively.
